# Ligand-induced conformational switch in an artificial bidomain protein scaffold

**DOI:** 10.1038/s41598-018-37256-5

**Published:** 2019-02-04

**Authors:** Corentin Léger, Thibault Di Meo, Magali Aumont-Nicaise, Christophe Velours, Dominique Durand, Ines Li de la Sierra-Gallay, Herman van Tilbeurgh, Niko Hildebrandt, Michel Desmadril, Agathe Urvoas, Marie Valerio-Lepiniec, Philippe Minard

**Affiliations:** Institute for Integrative Biology of the Cell (I2BC) CEA, CNRS, Univ. Paris-Sud, Université Paris-Saclay, 91198 Gif-sur-Yvette cedex, France

## Abstract

Artificial proteins binding any predefined “target” protein can now be efficiently generated using combinatorial libraries based on robust protein scaffolds. αRep is such a family of artificial proteins, based on an α-solenoid protein repeat scaffold. The low aggregation propensity of the specific “binders” generated from this library opens new protein engineering opportunities such as the creation of biosensors within multidomain constructions. Here, we have explored the properties of two new types of artificial bidomain proteins based on αRep structures. Their structural and functional properties are characterized in detail using biophysical methods. The results clearly show that both bidomain proteins adopt a closed bivalve shell-like conformation, in the ligand free form. However, the presence of ligands induces a conformational transition, and the proteins adopt an open form in which each domain can bind its cognate protein partner. The open/closed equilibria alter the affinities of each domain and induce new cooperative effects. The binding-induced relative domain motion was monitored by FRET. Crystal structures of the chimeric proteins indicate that the conformation of each constituting domain is conserved but that their mutual interactions explain the emergent properties of these artificial bidomain proteins. The ligand-induced structural transition observed in these bidomain proteins should be transferable to other αRep proteins with different specificity and could provide the basis of a new generic biosensor design.

## Introduction

Efficient methods have been recently developed to create artificial proteins binding specifically to almost any desired protein target. The most established strategy is to design and assemble a very large library of protein variants, in which all variants share a same architecture usually based on a natural protein “scaffold”^1,2^. Each library variant is characterized by the presence of random amino acid substitutions on its outside surface. New binding properties can emerge from this hypervariable surface, and although very rare in the initial library, specific binders can be selected by genetic sorting methods such as phage, ribosome or yeast display. It is now clear that these strategies, which bypasse immunization, are generally applicable, as illustrated by the fact that a large set of new proteins binding specifically to almost any predefined protein targets were obtained from several published libraries^1,3,4^. Those specific binding proteins based on alternative scaffolds are usually more efficiently produced and engineered than antibody-based chimeric proteins, that suffer from high aggregation propensity. Furthermore, specific binders from disulfide-free protein scaffolds can be efficiently used in reducing environments such as the cytoplasm of living cells.

Engineering of specific proteins based on robust scaffolds opens a range of new opportunities. Sophisticated multidomain proteins such as artificial receptors, sensors or switches could be designed by integrating tailor-made binding proteins into multi-modular constructions^5–7^.

In order to convert a specific binder into a specific bio-sensor, the target-binding domain must be integrated with a transducing component in such a way that the target-binding event will give rise to a measurable signal. The signal can result from environment-sensitive fluorescent probes chemically grafted near the binding site of the recognition unit^8–10^. Alternatively, the ligand-binding event can induce a structural transition detected in a second “reporter” domain. A simple head to tail fusion of recognition and reporter domains is often not adapted as it usually produces functionally-independent domains. Only few general solutions have been suggested to create such inter-domain coupling. For example, insertion of a peptide within the surface loop of an enzyme^11^ or inserting a whole protein into another unrelated enzyme were shown to generate new bi-active proteins with unexpected functional properties^12^. The coupling of the inserted and host domains can be further engineered or evolved to create new ligand responsive enzymes^13,14^. Structural transitions were also engineered by designing a bidomain protein in such a way that the folding of the two domains are mutually exclusive^15^. Specific “affinity clamps” have been further developed into protein^16^ or peptide^17^ specific biosensors by fusing each of the two peptide-binding domains to a fluorescent protein giving rise to reconstituted GFP fluorescence^16^ or to a ligand-dependent FRET signal^17^. Recently, new biosensors using computationally designed protein-binding domain and ligand-induced protein stabilization have been described^18–21^.

In this paper, we propose a new design of a multi-domain protein switch based on an open/closed bivalve-shell-like protein and we describe the experimental behavior of the resulting bidomain protein based on this design.

We used a recognition unit based on our previous work with αRep proteins. αReps are a family of artificial repeat proteins^2,22^ composed of a HEAT-like motif^23^. Each protein member of the αRep library has the same general architecture but is endowed with a unique binding surface made by the juxtaposition of hypervariable residues^24^. Specific αRep binders for a wide range of arbitrarily predefined target proteins with unrelated sequences and structures have been selected by phage display or protein complementation assay^25^. This suggests that the αRep library is a general source of specific reagents. The crystal structures of five αRep-target protein complexes (PDB ID: 4JW2, 4JW3, 4XL5, 4XVP, 4ZV6) clearly showed that the targets are bound, as expected, on the hypervariable concave face of the αRep fold. Given the versatile binding surface grafted on a precisely defined and very stable fold, a designed molecular switch based on αRep proteins may later be adaptable to other proteins of this family binding different unrelated targets. This prompted us to explore new molecular devices using αReps as building blocks.

The general design described here is based on the quaternary structure of one the previously described αRep proteins, named A3. This protein spontaneously forms a homodimer in solution^23^. In the dimeric structure, the binding concave surfaces made from the variable residues from each monomer face each other and the dimer resembles a closed bivalve shell. Although relatively tightly associated, we showed that the two monomers can dissociate in presence of another αRep that binds to the concave surface of A3. These results suggest that a synthetic bi-domain protein made by two A3 monomers linked together should fold as a closed bivalve, but could then be opened by a second distinct protein. This structural transition will *a priori* give rise to a large relative motion of the two linked domains. The opening and closing of a bidomain bivalve shell protein, consecutive to the binding of its ligand appear as a general way to create a conformational switch in this protein family and could later be used to create a range of αRep-based biosensors.

Despite this opening and closing possibility, it is difficult to predict precisely how such bidomain proteins will behave. For example, it is expected that covalently linking the two subunits of the A3 homodimer should reinforce the interactions between the two monomers and therefore should stabilize a closed form. But, would this closed bivalve still be able to open in order to bind an external ligand? Would it be possible to observe such a structural transition in αRep with other binding specificities? For example, how a chimeric protein made by an A3-domain and a different unrelated αRep domain would behave in solution? Would it give rise to a closed-bivalve? What will be the behavior of such a bidomain in the presence of ligands specific for each domain? The properties of such proteins are currently unpredictable and a better appreciation of these effects is clearly essential for the conception of multi-modular devices.

Our goal was to address experimentally these questions and to establish clear observations on the possible behavior of such proteins.

## Results

### Conception of the bidomain proteins

Previously described αReps A3^23^ and bGFPD^26^ were connected by a flexible linker (SGGGG)^2^, creating two bidomains named A3_A3 (homo-bidomain) and A3_bGFPD (hetero-bidomain) (Figure S1).

αRep A3 contains 4 internal repeats between an N-cap and a C-cap motif and its biophysical properties and crystal structure were previously described. The A3 homodimer dissociates in solution upon interaction with two bA3-2 and bA3–17 αReps that were selected from the library as specific binders for A3^24^. The interaction surface was determined using the crystal structure of the A3/bA3-2 complex (PDB ID 4JW2). bGFPD was selected from the αRep library as a specific binder for eGFP. It also contains 4 internal repeats and 2 caps.

### Conformational equilibrium for the bidomains in solution

**Figure 1:**
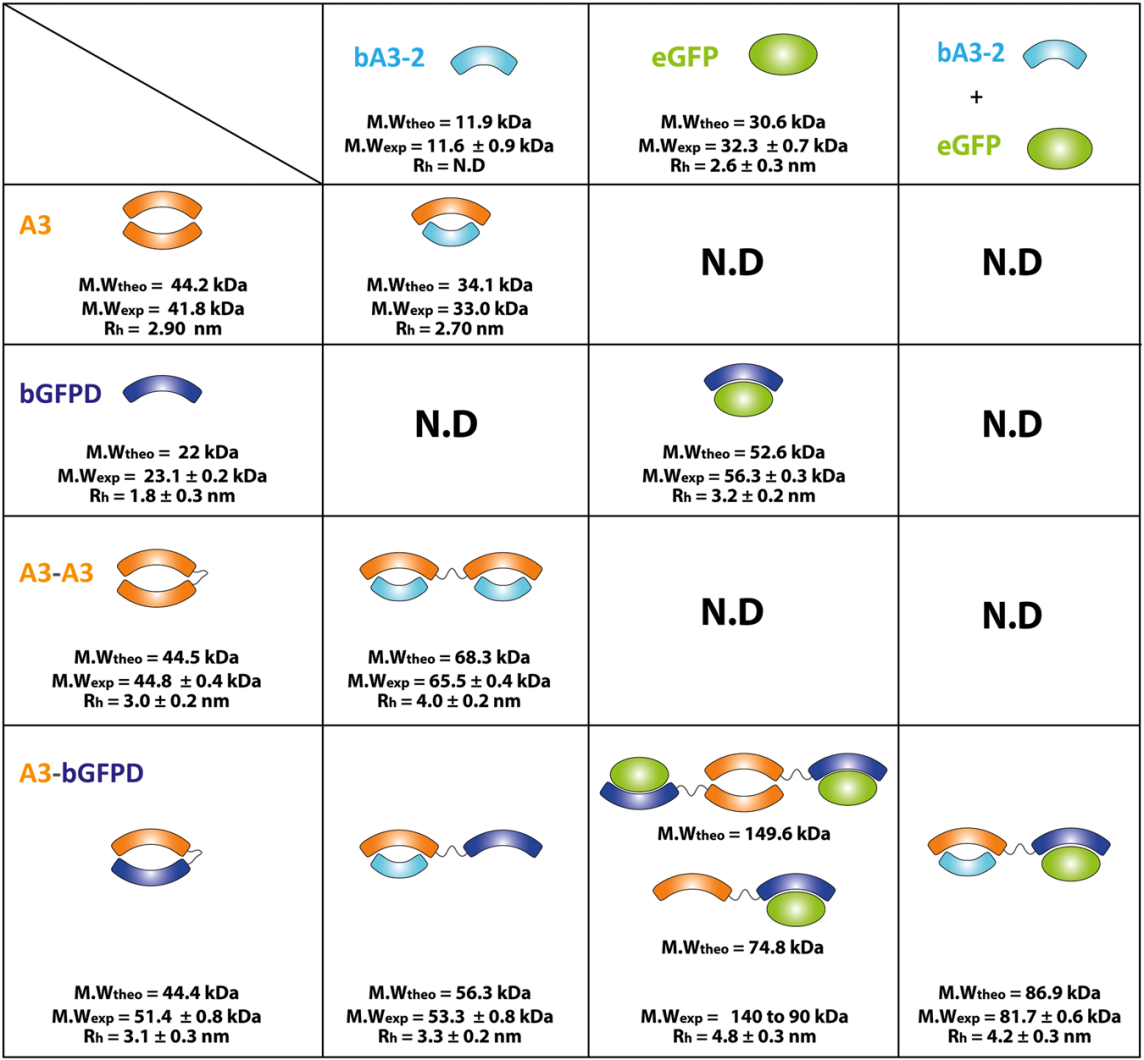
SEC-MALS characterization of the isolated αRep domains (A3 and bGFPD), bidomains (A3_A3 and A3_bGFPD) and their respective complexes. A3 is shown in orange, bGFPD in blue, the ligand bA3-2 in cyan and eGFP in green. For each complex, theoretical molecular weight (M.W_theo_) was calculated with the amino acid sequences using Expasy ProtParam Tool. Schematic representations of the complexes were deduced from experimental values. Experimental molecular weight (M.W_exp_) and hydrodynamic radius (Rh) were determined using the SEC-MALS data.

SEC-MALS experiments were performed with A3, bGFPD, A3_A3 and A3_bGFPD alone, and in the presence of their partners (Figs 1 and S2). The experimental molecular weights indicate that A3 is dimeric and bGFPD is monomeric in solution as previously reported^23,26^. As expected, the bidomain single chain, A3_ A3 is monomeric in solution, as the two linked domains interact together. The hydrodynamic radius of the bidomain A3_A3 (3.0 nm) is similar to the dimeric A3 (2.9 nm) indicating that the chimeric bidomain protein also adopts a closed bivalve conformation. SEC-MALS analysis of the A3_bGFPD construct yielded a broad and dissymmetric peak. Analysis of the higher elution volume fraction of the peak indicates a 44.8 kDa mass, which corresponds to A3_bGFPD monomer mass (Figure S2B). The mass corresponding to the top of the peak (51.4 kDa) suggests that the chimeric bidomain protein A3_bGFPD is in equilibrium between a monomeric and a dimeric form. Dimerization could be induced by the interaction of A3 domains from two distinct molecules. SEC-SAXS, a useful strategy to separate aggregates and/or oligomers from isolated proteins, has confirmed this conformational equilibrium (Figure S3 and Note S1).

### A3 and bGFPD interact weakly as isolated domains

Concerning the A3_bGFPD bidomain, a possible interaction between the A3 and bGFPD linked domains producing a closed monomeric form, was suggested from the SEC-MALS data. We therefore tried to quantify a possible interaction between free (non-linked) A3 and bGFPD domains using ITC: A3 was titrated with bGFPD and the isotherm clearly showed a weak interaction with a K_D_ of 4.1 ± 1.3 µM and a 1:1 stoichiometry. Analytical ultracentrifugation analyses confirmed the interaction of A3 with bGFPD with a measured K_D_ value of 3.3 ± 0.9 µM and a 1:1 stoichiometry. This suggests that a relatively weak affinity between the two isolated A3 and bGFPD monomers does exist, and is sufficient to stabilize a closed form when the two domains are linked into a single polypeptide chain.

### Each domain of each bidomain is still capable of interacting with its target

Upon the addition of one molar equivalent of each ligand (bA3-2 or eGFP) to the bidomains, changes in the SEC elution volumes and/or in the peak area were observed, indicating the formation of complexes of higher molecular weight. The stoichiometry of these complexes was determined by using the measured molar masses and molecular extinction coefficients. In both bidomains, each domain binds one molar equivalent of ligand (Figure S2C). Taken together, the SEC-MALS analyses reveal that both bidomains still bind their ligands (bA3-2 and eGFP) and that the two domains of one protein can bind their ligand simultaneously (Figs 1 and S2).

### New functional properties emerge from bidomain coupling

If these two bi-domains αRep adopt a closed form but are still able to bind their respective partners, then, a structural transition has to take place upon ligand binding and should be detected through its effects on the binding properties of the chimeric proteins. The interactions between the different partners were then quantified (Table 1). We first studied the isolated domains A3 and bGFPD with their respective targets, bA3-2 and eGFP using Isothermal Titration Calorimetry (ITC). As previously reported^24,26^, bA3-2 interacts with A3 in a 1:1 stoichiometric *ratio* with a K_D_ value of 4.5 ± 7 nM and eGFP interacts with bGFPD with a K_D_ value of 2.7 ± 2.0 nM. The interaction between two A3 monomers resulting in the A3 dimer has been measured using Analytical Ultracentrifugation (AUC) and a K_D_ value of 37 ± 6 nM (K_D A3/A3_) was determined (Table 1, Figure S4).

**Table 1:** Dissociation constants obtained for the different complexes. ^[a]^SPR data were obtained as described in Fig. 3, with partner 2 (analyte) in solution and partner 1 (ligand) immobilized on the sensor chip surface. K_D_ values were determined using the Langmuir model. Errors on the K_D_ values were obtained according to: ΔK_D_ = K_D_ * (Δk_on_/k_on_ + Δk_off_/k_off_). ^[b]^ITC experiments were obtained as described in Fig. 2. Partner 2 injected from the syringe in the cell, containing partner 1. ITC data were treated using a model assuming equivalent and independent sites. ^[c]^UCA experiments depicted in Figure S3. ^[d]^FRET experiments depicted in Fig. 5. FRET intensities plotted as a function of total Ligand were fitted as described in Material and methods section. ^[e]^Constants describing the intrinsic properties of the bidomains (K_intra A3_A3_ and K_intra A3_bGFPD_) defined in Figure S5.

Partner 1		Partner 2	K_D_ (nM)				
			SPR^[a]^	ITC^[b]^	UCA^[c]^	FRET^[d]^	Constant definition^[e]^
A3	*VS*	bA3-2	1.1 ± 0.1	4.5 ± 7.0			K_D1_
bGFPD		eGFP	2.3 ± 0.1	2.7 ± 2.0			
		BFP		5 ± 2.0			
A3		A3		n.a.	37 ± 6		K_D A3A3_
A3		bGFPD		4.1 ± 1.3 10^3^	3.3 ± 0.9 10^3^		K_D A3bGFPD_
A3_A3		bA3-2	46.0 ± 2.5	37 ± 7		623 ± 86	K_app1_
A3_A3 + excess bA3-17		bA3-2	—	9.7 ± 1.5			
A3_A3 + excess bA3-2		bA3-17	—	n.a.			
A3_bGFPD		bA3-2	—	77 ± 13		57 ± 10	K_app2_
A3_bGFPD		eGFP	12.5 ± 1.2	40 ± 4			
		BFP				134 ± 20	
A3_bGFPD + excess eGFP		bA3-2	—	21 ± 10			
A3_bGFPD + excess bA3-2		eGFP	—	8.9 ± 2.1			

### For A3_A3, apparent KD values depend on the bidomain’s conformation

**Figure 2:**
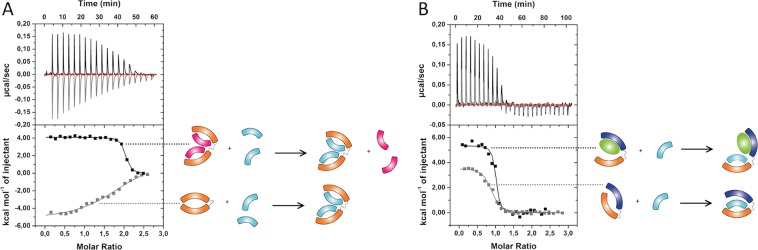
Competition binding experiments of the A3_A3 and A3_bGFPD bidomains pre-incubated with their respective ligands monitored by ITC. (**A**) ITC titration of a solution of A3_A3 (25 µM) either alone (in grey), or pre-incubated with an excess of the protein ligand bA3-17 (100 µM) (black) with bA3-2 (300 µM). These data indicate that bA3-2 can bind the bidomain with a stoichiometry of two bA3-2 ligands per bidomain (one ligand *per* A3 subunit); bA3-2 binds the bidomain with an increased apparent affinity in the presence of bA3-17 compared with the data obtained without bA3-17 pre-incubation. (**B**) ITC titration of monomeric A3_bGFPD (8 µM) alone (grey) or pre-incubated with an excess of the protein ligand eGFP (40 µM) (black) with bA3-2 (100 µM). bA3-2 binds the bidomain with a stoichiometry of one bA3-2 *per* bidomain (one ligand *per* A3 subunit) and an increased apparent affinity in the presence of eGFP.

A3_A3 was first titrated with bA3-2 by ITC. The stoichiometry (two bA3-2 for one A3_A3) (Table 1, Fig. 2A) indicates that each subunit is able to bind one ligand molecule, which confirms the SEC-MALS stoichiometry data. However, the K_D_ value of bA3-2 binding on A3_A3 (37 ± 6 nM) is higher than with A3 alone (without linker: 4.5 ± 7 nM), suggesting that the intramolecular link between the two binding sites stabilizes the closed form and induces a lower apparent affinity for an external ligand. The ITC data were reasonably well fitted with a model assuming equivalent and independent sites. It seems however clear that, if the two binding sites of the chimeric protein A3_A3 are nearly identical, the binding of a ligand on the first site will stabilize an open conformation of the bidomain structure and will facilitate the binding on the second site. The K_D_ determined by this experiment should therefore be considered as an apparent value (named K_app1_ in Figure S5), which averages the binding of two non-independent sites.

In a second experiment, the effects of the open/close equilibrium on ligand binding were probed. The idea was to “pre-open” the chimeric protein with an alternative partner before monitoring ligand binding. The αRep protein bA3–17 was previously characterized as a ligand for A3 with a lower affinity than bA3-2^24^. The A3_A3 bidomain was pre-incubated with one of its ligands and the second ligand was then added as a competitor. In this experiment, bA3–17 was not able to replace pre-bound bA3-2 as illustrated by the lack of signal in ITC. This result is in accordance with the known lower affinity of bA3–17 for A3, relatively to bA3-2 for A3. In the reverse experiments, bA3-2 was able to replace bA3-17 with a K_D_ value 9.7 ± 1.5 nM (Table 1, Fig. 2A). Thus, the apparent affinity associated to bA3-2 binding on A3_A3 actually increased when the A3_A3 protein was pre-incubated with another ligand. This suggests that A3_A3, when bound to a first protein ligand (with bA3–17 here), is in an open form while it is in a closed conformation before pre-incubation. In other words, the stability of the closed conformation contributes to lower its apparent affinity for bA3-2.

### For A3_bGFPD, binding of the ligands induces apparent positive cooperativity

According to the SEC-MALS data, the A3_bGFPD bidomain is in equilibrium between a monomeric and a dimeric form. SAXS experiments revealed that, below 8 μM, the A3-bGFPD bidomain is mainly monomeric. To measure the interaction of ligands on the monomeric form of A3_bGFPD, we therefore performed the following ITC binding experiment with a 8 μM solution of bidomain.

The binding of bA3-2 or eGFP to A3_bGFPD was measured first individually and then sequentially. Stoichiometric interactions with A3_bGFPD were observed with K_D_ values of 77 ± 13 nM for bA3-2 and 40 ± 4 nM for eGFP (Table 1, Fig. 2B). Once again, each ligand is able to interact with its partner domain, but the apparent affinities are lower than those observed for the individual domains. This suggests that the covalent link strengthens interactions between the two domains and stabilizes a closed form for the bidomain.

Following pre-incubation of A3_bGFPD with eGFP, bA3-2 could bind with a K_D_ value of 21 ± 10 nM (Table 1). When pre-incubated with bA3-2, eGFP binds with K_D_ value of 8.9 ± 2.1 nM. Both pre-incubations could be a way to convert the closed conformation of the bidomain into an open state thereby facilitating the binding of the second ligand. Yet, the measured affinity of bA3-2 and eGFP for the A3_bGFPD following pre-incubation is still lower than the one for A3 or bGFPD alone, suggesting a steric hindrance of both ligands for the interaction with their respective domain’s surface.

Overall, these ligand effects can be considered as positive heterotropic cooperativity between the two sites of the bidomain: the binding of the first ligand facilitates the binding of the second one. These binding properties directly support the existence of ligand induced conformational transitions in both chimeric proteins.

### Kinetics of ligand interactions reveal ligand-induced avidity effects

The kinetics of ligand association and dissociation with the two bidomains were analyzed by Surface Plasmon Resonance (Table 1).

The K_D_ values calculated from the association and dissociation fitting curves using the Langmuir Model were 2.3 ± 0. 1 nM for circulating bGFPD with immobilized eGFP and 1.5 ± 0.2 nM for circulating A3 with immobilized bA3-2. The maximum concentration of A3 in this experiment was 4 nM and at this concentration, A3 is more than 90% monomeric according to the dimerization constant K_D A3/A3_ determined by AUC (37 ± 6 nM). We thus measured the affinity of bA3-2 for the monomeric form of A3, which is, as expected, higher than for the A3 dimer.

**Figure 3:**
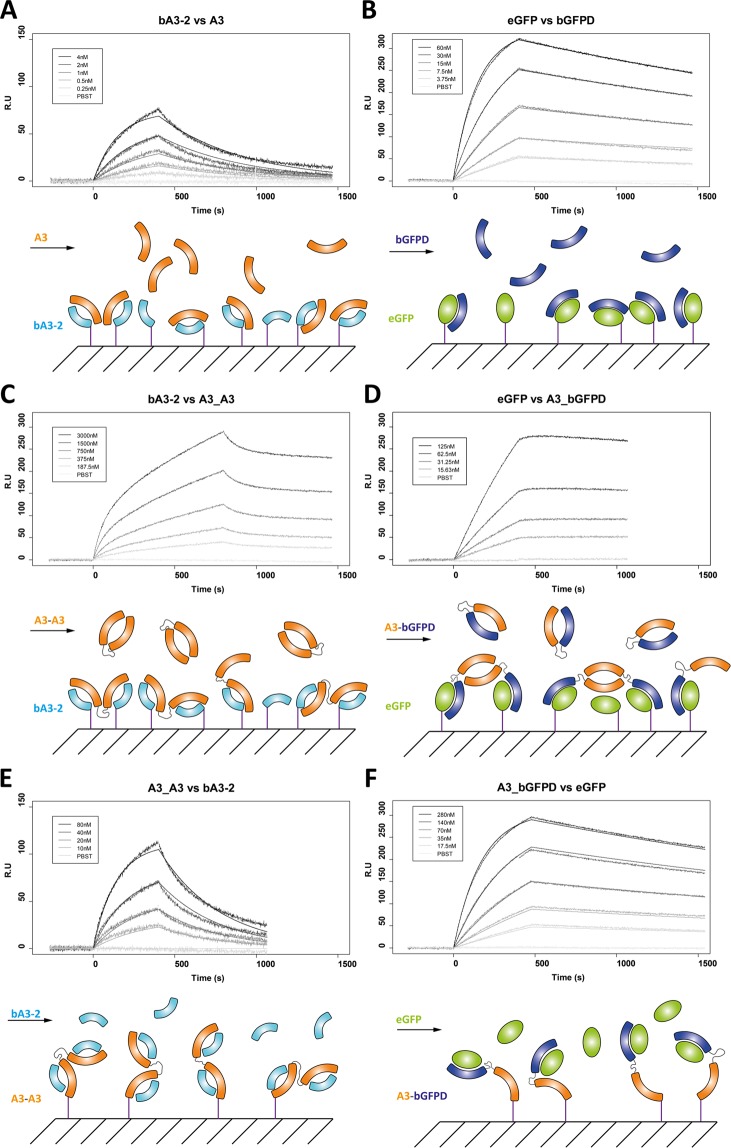
Surface Plasmon Resonnance analysis of interactions of αReps (A3 and bGFPD) or bidomains (A3_A3 and A3_bGFPD) with their respective partners. His-tagged proteins are immobilized as ligands on Nickel-loaded sensor chips. Proteins used as analytes have no His-tag. A3 has a C-terminal Strep-Tag, bA3-2 a C-terminal Twin Strep-Tag, and the His-tag of the eGFP, A3_A3 and A3_bGFPD were cleaved by the TEV protease. (**A**) Five concentrations of monomeric A3 protein (4 nM, 2 nM, 1 nM, 0.5 nM, 0.25 nM) were applied to 5 channels containing immobilized bA3-2. The data were fitted using the Langmuir model with k_on_ = 1.3 ± 0.1 10^6^ M^−1^. s^−1^, k_off_ = 1.9 ± 0.1 10^−3^ s^−1^ and K_D_ = 1.5 ± 0.2 nM. (**B**) Five concentrations of bGFPD (3.75; 7.5; 15; 30; 60 nM) were applied to immobilized eGFP. The data were fitted using the Langmuir model with k_on_ = 1.1 ± 0.1 10^5^ M^−1^. s^−1^, k_off_ = 2.5 ± 0.1 10^−4^ s^−1^ and K_D_ = 2.3 ± 0.2 nM. (**C**) Five concentrations of A3_A3 (188; 375; 750; 1500; 3000 nM) were applied to flow cell with immobilized bA3-2. (**D**) Five concentrations of A3_bGFPD (15; 31; 62; 125; 250 nM) were applied to immobilized eGFP. The dissociation is slower for bidomains (C and D) than for the isolated domains, which is characteristic of an avidity effect. The curves could not be fitted using the langmuir model. (**E**) Four concentrations of bA3-2 (10; 20; 40; 80 nM) were applied to immobilized A3_A3. The data were fitted using the Langmuir model with k_on_ = 5.8 ± 0.1 10^4^ M^−1^. s^−1^ and k_off_ = 2.7 ± 0.1 10^−3^ s^−1^ and K_D_ 46.0 ± 2.5 nM. (**F**) Five concentrations of eGFP (17.5; 35; 70; 140; 280 nM) were applied to immobilized A3_bGFPD. The data were fitted using the Langmuir model with k_on_ = 2.0 ± 0.1 10^4^ M^−1^. s^−1^ and k_off_ = 2.5 ± 0.1 10^−4^ s^−1^ and K_D_ = 12.5 ± 1.2 nM.

When the bidomains are immobilized on the chip, the profile of the kinetics of ligand association and dissociation obtained with either bA3-2 (Fig. 3E) or eGFP (Fig. 3F) as circulating analytes are comparable to those observed when each isolated domain is immobilized (Fig. 3A,B).

Bidomains A3_A3 and A3_bGFPD were then used as circulating analytes on bA3-2 and eGFP immobilized ligands. The curves could not be fitted using simple models, but, clearly, the creation of chimeric proteins affects both association and dissociation phases. For both complexes bA3-2/A3_A3 and eGFP/A3_bGFPD (Fig. 3B,D), a slower association was observed for the chimeric bidomain protein relatively to their non-chimeric equivalents (bA3-2/A3 and eGFP/bGFPD complexes) (Fig. 3A,C). This slower association is expected as an equilibrium exists between the open and closed states of the bidomain. The slower dissociation was more unexpected, but can be attributed to a ligand-inducible avidity effect for both bidomains. Indeed, the A3_A3 bidomain, once opened, can bind simultaneously two immobilized bA3-2 (Fig. 3B, bottom). Similarly, when the A3_bGFPD bidomain binds one eGFP ligand immobilized on the chip, its second domain (A3) is released and its dimerization is enhanced with an A3 domain from another bidomain present in the analyte solution, or bound on the surface. Thus, it induces an avidity effect for the second eGFP binding site with a very slow dissociation from the chip (Fig. 3D, bottom).

### Structural analyses of the bidomains are in agreement with the interactions experiments

**Figure 4:**
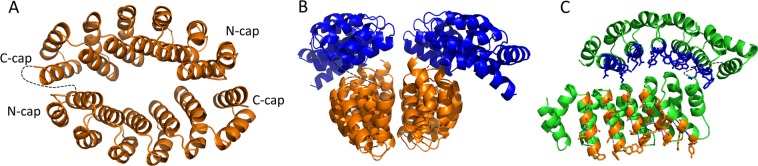
Crystal structures of the bidomain proteins A3_A3 and A3_bGFPD. (**A**) X-Ray structure of the protein A3_A3. The linker is not visible in the bidomain due to its flexibility. (**B**) X-Ray structure of A3_bGFPD. A3 domains are in orange and bGFPD domains are in blue. Two chimeric molecules A3_bGFPD dimerize through their A3 domain (orange). (**C**) Representation of the variable and constant residues in the structure of A3_bGFPD. Constant residues are in green and variable residues are in orange for the A3 domain and in blue for the bGFPD domain. In the crystal, variable residues (in blue) of the bGFPD domains are in contact with constants residues located on the A3 domain to form a T-shape object. In all these structures, the linker is schematized with grey dashes.

Structural studies of the two bidomains were undertaken in order to have a better understanding of their behavior. The crystal structures of the two bidomains were determined with a resolution of 1.94 and 2.55 Å for A3_A3 and A3_bGFPD respectively. In neither structure electronic density was observed for the linker, which was expected as it was designed to be totally flexible. A3_A3 crystallizes with one A3 subunit in the asymmetric unit. SDS-PAGE of dissolved crystals showed that they contained the intact fusion protein (Figure S6). The crystal structure of A3_A3 (PBD ID 6FT5) is comparable to the previously described dimeric A3 (PDB ID 3LTM, PDB ID 3LTJ) revealing that the linker does not affect the interface between the two A3 domains (Fig. 4A). The structure analysis of the A3 dimer previously solved showed that the two N-cap/C-cap are involved in the interface between the two subunits of the dimer^23^. This suggests that this interface between caps of each monomer contributes to the dimer association. As the conformation of the A3_A3 bidomain is fully superimposable to the A3 dimer, the interactions involving the N-cap and C-cap are also present in the bidomain and favor the close conformation.

The A3_bGFPD (PBD ID 6HWP) bidomain crystals have a single A3_bGFPD in the asymmetric unit. The structure shows an open conformation but the two domains are in contact: the loop linking the consecutives repeats of the A3 domain interact with the GFP binding surface of the second bGFPD domain. Importantly, the crystal symmetry generates a dimer with a neighbor molecule *via* its A3 subunit. This dimer is identical to the A3 dimer and to the A3_A3 bidomain protein. SEC-MALS and SAXS results indicate the presence of an equilibrium between a monomeric and a dimeric form of the bidomain and at the concentration used for crystallization, the dimeric form is favored. For the dimer observed in the crystal structure (Fig. 4B), two distinct interfaces are observed. The first one is the dimerization interface established between the two A3 domains within a dimer, and the second involves the A3 domain and the bGFPD domain of the same molecule. The dimerization interface between two A3 domains (1170 Å^2^) is higher than the interface (638 Å^2^) between the A3 and bGFPD domain of the same molecule. Furthermore, these two interfaces involve different types of residues. The dimeric interface between A3 domains is established by contact between their variable side chains. In the interdomain interface, the variable residues of the bGFPD domain are in contact with a non-variable part of the surface of A3 (Fig. 4C). Since the non-variable surfaces of the A3 and bGFPD domains are identical, these A3/bGFPD interactions, if stable enough, should also take place between two bGFPD molecules. This would lead to an oligomerization of the bGFPD protein, which is not observed in solution. Thus, the interaction between the bGFPD and A3 domain observed in the crystal structure may not reflect the interdomain interactions taking place in solution, for A3_bGFPD, but is rather a secondary consequence of crystallization of the dimeric form.

A SEC-SAXS experiment has been used to determine the conformation of A3_bGFPD in solution. The successive scattering patterns recorded at the end of the elution peak (Figure S3A), corresponding to the monomer, are averaged. The resulting curve (Figure S3C,D) shows that A3_bGFPD is rather compact in solution (R_g_ = 26.8 Å, D_max_ = 90 Å), and could adopt different possible geometries of the closed forms.

### Conformational transition consecutive to Ligand binding events can be detected by FRET

**Figure 5:**
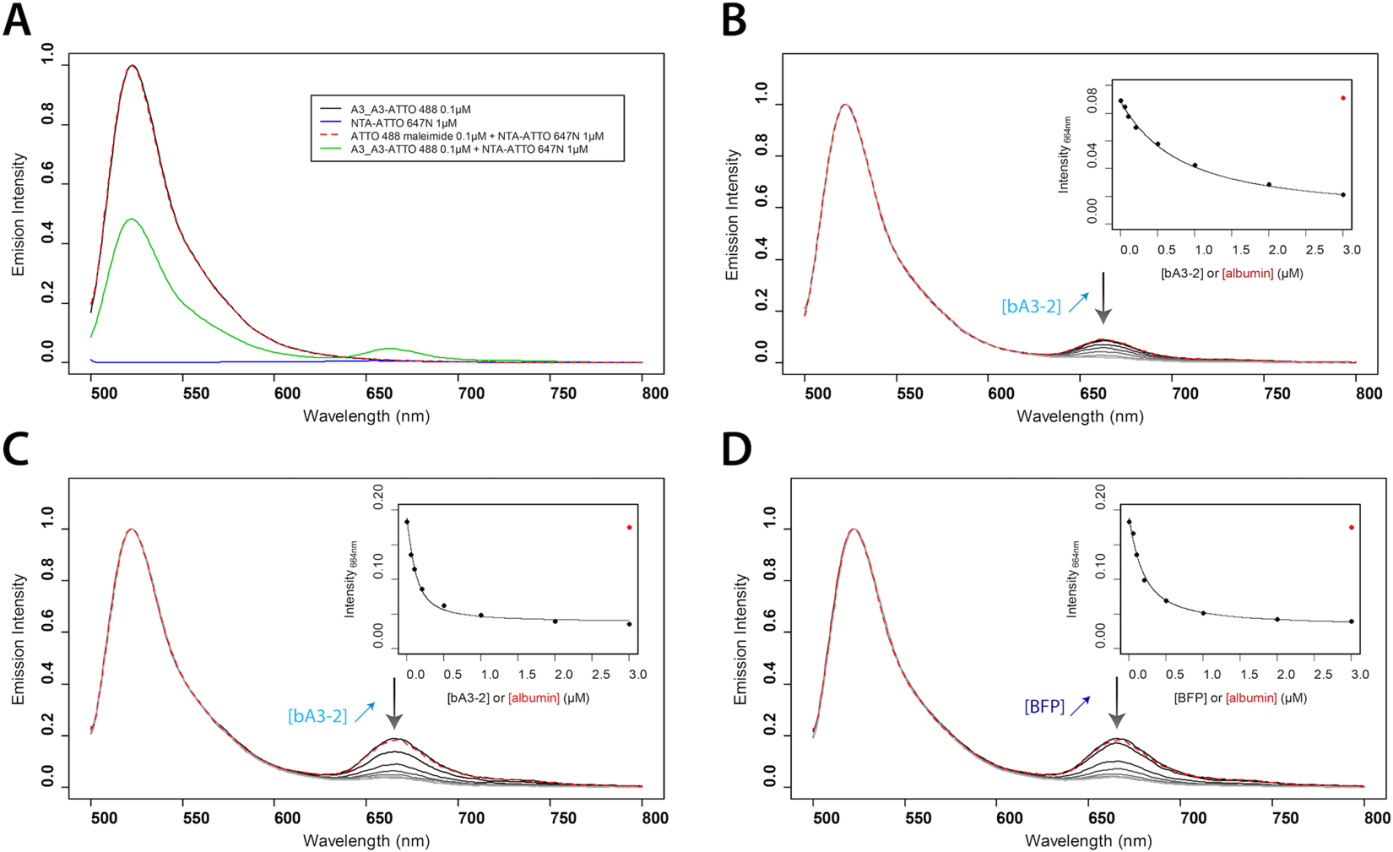
FRET measurements assessing conformational changes of the bidomains A3_A3 and A3_bGFPD. A3_A3 or A3_bGFPD bidomains are conjugated with two dyes, NTA-ATTO 647 N at the N-Terminal His-tag (Acceptor) and ATTO 488 maleimide covalently attached on a C-terminal cysteine (Donor). 0.1 µM ATTO 488-bidomain were mixed with 1 µM NTA-ATTO 647 N. Ligands bA3-2 and BFP were used at concentrations of 0.05, 0.1, 0.2, 0.5, 1, 2 and 3 µM without His-Tag. Albumin was used as a control at a concentration of 3 µM. All emission spectra were recorded with an excitation at 488 nm. (**A**) Fluorescence emission spectra of A3_A3-ATTO 488 (0.1 µM) (black line), NTA-ATTO 647 N (1 µM) (blue line), ATTO 488 maleimide (0.1 µM) mixed with NTA-ATTO 647 N (1 µM) (red dashed line) and a mixture of A3_A3-ATTO 488 (0.1 µM) and NTA-ATTO 647 N (1 µM) (green line). The emission signal at 662 nm, only for the dually labelled protein suggests fluorescence transfer between the two dyes in the bidomain. (**B–D**) Normalized emission spectra of the dually labelled A3_A3 (**B**) and A3_bGFPD (**C, D**) with different concentrations of bA3-2 (**B, C**) and BFP (**D**). The emission peak of ATTO 647 N (664 nm) decreases for both bidomains in the presence of increasing concentrations of bA3-2 or BFP. Albumin (red dashed line) has no effect on the fluorescence. (Inset) Emission signal at 664 nm, in the presence of increasing ligand concentrations (bA3-2 or BGP) was fitted as a function of total ligand concentration (K_D_ values reported in Table 1).

In order to monitor if binding events could give rise to a measurable fluorescence output, both bidomains were coupled with a FRET pair of fluorescent dyes^27^. A unique C-terminal cysteine was covalently grafted with an “ATTO 488 maleimide” dye used as FRET donor and the N-terminal His-tag was coupled with a “NTA-ATTO 647 N” dye used as a FRET acceptor. For both bidomains, (Figs 5A and S7C) a FRET signal (quenching of the donor fluorescence intensity and sensitization of the acceptor fluorescence intensity) between the two dyes is observed, as expected since the closed conformation of the bidomain brings the two dyes into close proximity. The labeled bidomains were titrated with their respective ligands (bA3-2 for both bidomains and BFP for A3_bGFPD). The blue fluorescent variant BFP was chosen as an alternative ligand of bGFPd, rather than eGFP used in the other experiments, in order to avoid the overlap in the absorbance spectra with eGFP^28^ (Figure S7A). Control experiments indicated that the affinities of BFP and eGFP for bGFPD are comparable, as assessed by ITC (Figure S7B). In all cases, the FRET sensitized acceptor emission signal at 664 nm decreased with increasing ligand concentrations (Fig. 5B–D), because ligand binding pushes the chimeric proteins into an open conformational transition consecutive to ligand binding and thereby increases the donor-acceptor distance, which leads to a lower FRET efficiency and a concomitant decrease of acceptor emission intensity. For A3_bGFPD, the K_D_ values obtained for both ligands are in the same range as the values obtained by ITC, while for A3_A3, the K_D_ value is significantly higher than the one determined by ITC (Table 1). However, these two experiments do not monitor the same binding events: in the ITC experiment the binding of each one of the two ligands on the protein is monitored, whereas in the FRET experiment the signal monitors the binding of the first ligand molecule that opens the bidomain and consequently disrupts the FRET between the donor and acceptor. As observed with A3_bGFPD, the FRET signal is abolished upon binding of the first ligand molecule. This suggests that for, the A3-A3 bidomain, the effect of the second bound ligand on the FRET signal is very limited and do not contribute to the estimation of the apparent K_D_. Overall, these fluorescence data clearly show that the conformational changes suggested by the ITC and SPR results can be directly monitored by FRET variation with donor/acceptor pairs located at each extremity of the bidomain proteins.

## Discussion

### Chimeric bidomain αReps adopt a closed form

The SEC-MALS and SAXS results clearly show that, at low micromolar concentration both bidomains adopt a closed bivalve conformation. This was expected for A3_A3: when not linked together the two A3 monomers dimerize with relatively high affinity (K_D A3/A3_ = 37 nM, ultra-centrifugation data, Table 1), and in the A3_A3 bidomain, their mutual interactions are further stabilized by their proximity. The closed form of A3_bGFPD was not anticipated. Indeed, since the bGFPD domain was initially selected to interact specifically with eGFP, it was *a priori* not expected to bind the A3 domain. Nonetheless, ITC and AUC experiments revealed that these two unrelated domains do interact, although with a low affinity (K_D A3/bGFPD_ ≈4 µM). However even this relatively weak affinity between domains is sufficient to take place predominantly within a bidomain protein. This fortuitous and relatively weak interaction between A3 and bGFPD moieties may in fact be due to the interactions between the N-cap of the A3 domain and the C-cap of the bGFPD domain. All αReps share the same C-cap repeat. The A3 dimer structure clearly indicates that the specific sequence of the A3 N-cap can interact with the common C-cap repeat and this could contribute to the observed affinity.

### Ligand induced structural transitions in multi-domains αReps result in functional effects

The binding surfaces of the bidomain are covering the interior of the bivalve-like structure. The closed forms are therefore not structurally compatible with the presence of bound partners. However, for both bidomains, each domain can still bind its cognate partners, when proteins undergo a structural transition upon ligand binding.

**Figure 6:**
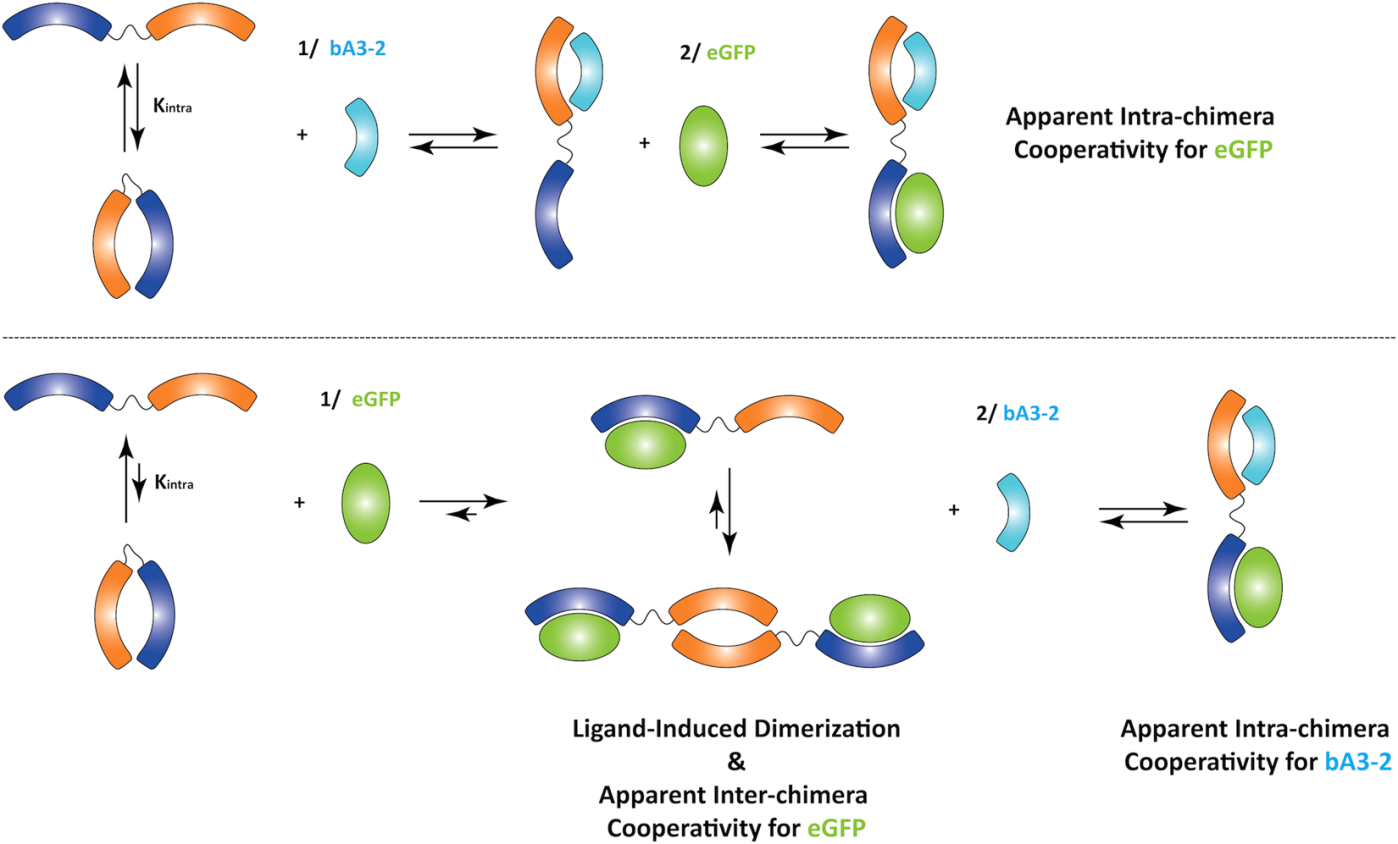
Model for the binding of the two A3_bGFPD ligands whether bA3-2 or eGFP binds first. The binding of the eGFP tends to form a dimeric bidomain by releasing A3 domains in a ligand-induced cooperativity process. When ligands are in excess, the complexes are more prone to form heterotrimers, each domain binding its respective ligand (A3/bA3-2 and bGFPD/eGFP). Such a process could be generalized to develop biosensors with any αRep binding a specific target of interest.

The *equilibria* between the open and closed conformations for these bivalve proteins modulate their binding affinities for their respective ligands in a cooperative process. This is particularly clear for the A3_A3 protein, which still binds its ligand although with an apparent lower affinity than A3. This low affinity increases if the bidomain protein is pre-opened with another competitive ligand, which is characteristic of positive homotropic cooperativity (Fig. 6A). Similarly, the results suggest that positive heterotropic cooperativity is observed with the A3_bGFPD protein when the two ligands (bA3-2 and eGFP) bind together (Fig. 6B). Indeed, when eGFP is bound to the bidomain first, the affinity of bA3-2 is increased by a factor of 3.6. When bA3-2 is bound first, the affinity of eGFP increases by a factor of 4.4. However, in neither case do the pre-opened forms recover the affinity observed for the same ligand on the monomeric domain. This suggests that, if the open/closed equilibrium contributes to change the affinity of concatenated αRep domains, the open form cannot bind the ligand as efficiently as the individual domain.

### Ligand induced dimerization

SEC-MALS and SPR experiments show that it is possible to induce the dimerization of the A3_bGFPD protein *via* the A3 domains by adding eGFP. When eGFP is immobilized, this dimer of bidomains can therefore bind two anchor points on the chip, thus inducing an avidity effect. This process can considerably slow down the dissociation of the chimeric proteins from the binding surface. This “cooperavidity” effect can be expected for any chimeric protein made from a dimerization domain shielded by a ligand-binding domain. The ligand-binding event releases the dimerization domain, and if the ligand density is sufficient, this in turn induces an avidity effect. The closed form, dominant in absence of the target, should minimize accessibility of the binding surface but dimerization consecutive to binding should strongly favor retention of the bound bidomain on a surface specifically where two target molecules are in close proximity. This effect could be potentially useful for example to enhance contrast in cellular imaging.

### Potential applications of complex bidomains such as sensors and switches

The experiments described here were conducted with specific αRep domains (A3 and bGFPD) but the underlying principles can be more generally useful. αRep binders for various unrelated proteins have already been generated^24,29–32^. The binding surface is invariably located, as expected, on the randomized surface. All αReps share the same general fold and could therefore form a closed bivalve like bidomain structure when fused to an A3 or A3-like closing domain. The “open/closed bivalve effect” described here could therefore possibly be observed on many other αRep-based recognition unit.

As shown in the FRET assays for these two “model” bidomains, it is clearly possible to monitor the open/closed conformational change into a variation of fluorescence signal correlated to ligand binding. Such a closed/open transition in a bivalve-like protein could later prove useful as a general way to transduce the target-binding event into a measurable quantitative readout. Here, it will be possible to tailor chimeric proteins with domains that bind a target of choice, which is a substantial improvement over existing sensors.

Moreover the cooperative effects observed between the binding partners of a chimeric protein made from two different αReps with different specificities could also be a basis to design future molecular switches. The structural transition induced by the ligand of one domain can enhance the binding on the second domain of a different, unrelated partner.

## Materials and Methods

### Conception of the proteins

The αRep proteins are mainly composed of repeated sequences and therefore molecular biology procedures such as cloning based on PCR can be difficult due to the repeated codon sequences. This is further enhanced in sequences of bidomain proteins based on αRep. In order to simplify the constructions steps, two distinct DNA sequences coding for the same αRep A3 protein were used. The first one is the gene directly obtained from the αRep library (A3). The second one is a synthetic gene (*a3s*) previously described^33^. The DNA sequence *a3s* was designed with different codons at equivalent positions in consecutive repeat and therefore could be easily amplified by PCR.

The sequence coding for TEV-A3 and a linker (SGGGG)_2_ were inserted ahead of the multiple cloning site in PQE81L using circular polymerase extension cloning (CPEC). In this step a new pair of unique cloning sites (BamH I/Hind III) was inserted just after the sequence of the linker. These sites were used to insert the *a3* sequence using classical restriction sites cloning. The gene coding to bGFPD was cloned using the same cloning site (BamH I/Hind III).

Gene construction of both bidomains *A3_A3* and *A3_bGFPD* are described more precisely in Supplementary Figure S1. His-tag constructs were obtained in pQE80L or pQE81L plasmids (Qiagen). N-terminal Twin-Strep-Tag^®^ constructs (IBA technology) were obtained from PQE80L in which the His-tag was removed.

### Protein expression and purification

Expression and purification of His-tagged proteins were performed as described^23,24,26^. *E. Coli BL21-Gold (DE3) Competent Cells (Agilent Technologies)* were transformed with the plasmid coding for the different proteins. Cells were grown at 37 °C in 2YT medium containing 200 µg.L^−1^ ampicillin until the OD_600nm_ reached 0.6. Protein expression was induced by addition of 1 mM IPTG and the cells were further incubated for 5 h at 37 °C. The cells were harvested, suspended in 50 mM sodium phosphate pH7.5, 150 mM NaCl (PBS), submitted to three freezing/thawing cycles, treated with DNase I for 30 min and sonicated. The His-tagged proteins were purified using nickel-affinity chromatography (Histrap™ FF crude 5 mL, GE Healthcare). The His-tag A3-A3 and A3-bGFPD was cleaved by TEV protease for some SPR experiments.

Twin-Strep®-tagged proteins (bA3-2, eGFP and BFP) were also purified using a StrepTactin Sepharose affinity chromatography (StrepTrap™ HP 5 mL, GE Healthcare); both samples obtained from this first step purification were then submitted to a size exclusion chromatography.

### Size Exclusion Chromatography - Multi-Angle Light Scattering (SEC-MALS)

Purified proteins (100 µL, 2 mg.mL^−1^) were loaded on a Superdex 200 10/300GL increase (GE Healthcare) equilibrated in PBS at a 0.5 mL.min^−1^ flow rate (Shimadzu HPLC system). Detection was performed using a MiniDAWN TREOS multi-angle light scattering detector and an Optilab T-rEX differential refractometer (Wyatt Technology). Absolute molar masses were determined with the Astra 6.1 software, using a differential index of refraction (dn/dc) value of 0.183 mL.g^−1^. Hydrodynamic radius was determined using the WyattQELS (Quasi-Elastic Light Scattering) module.

### Isothermal titration calorimetry

The binding parameters were examined either with an ITC 200 microcalorimeter (MicoCal, Malvern) or with a VP-ITC (MicoCal, Malvern). For the measurement monitored with the ITC 200, 2 µL aliquots of the titrant (generally bA3-2 and eGFP) at 300 µM were injected from a computer-controlled 40 µL microsyringe at intervals of 180 s into the solution of target (25 µM) dissolved in PBS (stirring at 700 rpm). For the measurement monitored with the VP-ITC, 10 µL aliquots of the titrant at 100 µM were injected from a 250 µL microsyringe at intervals of 240 s into the solution of target (8 µM) with or without 40 µM competitor) in PBS (stirring at 350 rpm). The data were integrated and analyzed using the MicroCal Origin software provided by the manufacturer according to the one-binding-site model.

### Surface Plasmon Resonance

Surface plasmon resonance was measured using a ProteonTM XPR36 instrument (Bio-Rad). Immobilized proteins contain a N-terminal His-tag. Circulating proteins have no His-tag (TEV-cleaved for the bidomain or N-terminal Twin-Strep-tag for bA3-2 and eGFP). All measurements were performed in PBS containing 0.05% Tween20 at a flow rate of 50 µL·min^−1^. ProteOn HTG sensor chip (Bio-Rad) were used to immobilized αRep proteins (bA3-2 and eGFP) at two densities on two of the six channels chip following the Tris-NTA / His-tag coupling protocol. Five concentrations (from 0 to 3 µM) of purified proteins (A3, bGFPD, A3_A3 and A3_bGFPD) were tested. Signals were corrected with the uncoated reference channel and fitted with the Proteon Manager software by Langmuir model. The final figures were obtained using RStudio software.

### Analytical Ultracentrifugation

All sedimentation velocity experiments were performed on an analytical ultracentrifuge XLI (Beckman Coulter, Palo Alto, USA) with an An-50 Ti rotor at 20 °C equipped with a fluorescence detection system (AVIV Biomedical, Lakewood, NJ). Sedimentation velocity experiments were done in two-channel 12 mm path-length Epon charcoal-filled centerpieces. A3 protein was labeled with Monolith NT.115 Protein Labeling Kit BLUE NHS (Nanotemper Technologies). Samples were prepared by dilution of concentrated stocks in PBS, 0.1 mg.mL^−1^ of bovine serum albumin (BSA). For protein sample from 1 nM to 60 nM, only labeled protein was used; for higher concentrated samples labeled protein (60 nM) was mixed with unlabeled protein. 400 μL of A3 samples were centrifuged at 42,000 rpm (128,297 g). Sedimentation profiles were collected every 5 min and analyzed as described^34–36^.

### Small Angle X-Ray Scattering

SEC-SAXS data were collected with A3_bGFPD samples at the BM29 line at the ESRF, Grenoble, with a size-exclusion HPLC column (Agilent Bio sec-3) online with a SAXS measuring cell (a 1.5 mm diameter quartz capillary in an evacuated sample chamber) and the data analysis is detailed in Note S1.

Other SAXS experiments were performed on an in-house SAXS instrument (Brüker Nanostar; λ = 1.54 Å). 30 µl of concentrated solutions (0.5 mg mL^−1^ ≤ c ≤ 8.0 mg mL^−1^) of A3_bGFPD were placed in a quartz capillary thermalized cell inserted into an evacuated sample chamber. SAXS data were analyzed using the program PRIMUS (https://www.embl-hamburg.de/biosaxs/primus.html).

### Crystallization, structure determination and refinement

All crystallization experiments were carried out at 293 K using the vapor diffusion method. Initial crystallization screening was done using commercially available kits (Qiagen Classic, MB Class I, PEG I, JCSG + ). The two bidomains crystallized under several different conditions. For A3_A3, the optimized crystallization conditions are 0.1 M Sodium-citrate pH 5.6, 2.4 M Ammonium sulfate. For A3_bGFPD best crystals were obtained in 1.6 M Tri-Sodium citrate pH6.5. Crystals were flash-frozen by soaking into mother liquor supplemented with 30% glycerol as cryoprotectant for A3_A3 and 3.8 M malonate for A3_bGFPD. Diffraction data were collected at 100 K on beamline ID29 at the ESRF synchrotron using a PILATUS 6 M detector. The images were analyzed with the XDS program^37^. The structures were solved by molecular replacement using PHASER^38^ implemented in the CCP4 program suite^39^. αRep A3 structure, PDB ID 3LTJ^23^, was used as search model. The experimental map was improved by solvent modification using the program DM^40^. The initial models were completed by interactive and manual model building with the program COOT^41^. Refinement of structures was performed using REFMAC^42^.

The crystal structure of A3_A3 at 1.94 Å resolution was refined to R and R_free_ crystallographic factors of 17.48**%** and 22.21**%** respectively (Table S1). The crystal structure of A3_bGFPD at 2.55 Å resolution was refined to R and R_free_ crystallographic factors of 23.59**%** and 26.24**%** respectively (Table S1).

### Functionalization with fluorescent dyes

A C-terminal cysteine was added by directed mutagenesis on A3_A3 and A3_bGFPD. Proteins were reduced with an excess of DTT removed using Zeba Micro Spin desalting columns (Thermo Fisher). A 10 mM solution of ATTO 488 maleimide (Sigma) was prepared in anhydrous DMSO and mixed with the proteins samples in PBS (10 molar equivalent of dye per protein). The mixtures were incubated overnight at 4 °C in the dark. Excess of ATTO 488 maleimide was washed off using Zeba Micro Spin desalting columns. ATTO 488 concentrations were determined by absorbance measurements at 501 nm using a molar absorptivity of 90 000 M^−1^ cm^−1^.

Each ATTO 488-labelled bidomain was then conjugated with NTA-ATTO 647 N (https://www.atto-tec.com) via the N-terminal His-tag. 10 molar equivalents of dye were added to 0.1 µM of ATTO 488-bidomain and incubated for 5 min in the dark.

### FRET binding assays

FRET assays were performed on a SAFAS (Monaco) flx-Xenius V2 spectrofluorometer. Emission spectra were measured from 500 nm to 800 nm while exciting the samples at 488 nm. Spectra were recorded using a 1 nm step, a 10 nm bandwidth for both channels, an averaging time of 0.1 s and a PMT voltage of 500 V. Successive aliquots of bA3-2, BFP or albumin (without His-tag) were added in the cuvette containing 0.1 µM of labelled bidomain. The solutions were incubated for two minutes at room temperature in the dark before measuring each spectrum. Fluorescence emission intensities at 664 nm plotted as a function of total ligand concentration were fitted according to the following equation using the Origin program:

$$I={I}_{min}+{[{\rm{\Delta }}I]}_{max}\frac{[n({[P]}_{0}+{[L]}_{0}+{K}_{D})-\sqrt{{(n{[P]}_{0}+{[L]}_{0}+{K}_{D})}^{2}-4n{[P]}_{0}{[L]}_{0}}\,]}{2n{[P]}_{0}},$$

where [P]_0_ and [L]_0_ are respectively the total protein and ligand concentrations; ΔI_max_ = I_0_ − I_min_ with I_0_ the intensity in the absence of ligand, and I_min_ the asymptotic value for I; I_min_ and K_D_ are the calculated values from the fitting equation. The equation derives from the definition of the dissociation constant K_D_ as a function of total ligand and protein concentrations, assuming n independent and equivalent ligand binding sites on the protein. The final figures were obtained using RStudio software.

## Supplementary information

Supplementary Information

## Data Availability

All data generated or analyzed during this study are included in this published article (and its Supplementary Information files).
