# Supplementary material for: Ligand-induced conformational switch in an artificial bidomain protein scaffold
Source: Sci Rep. 2019 Feb 4;9:1178. doi: 10.1038/s41598-018-37256-5 (PMC6362204; doi:10.1038/s41598-018-37256-5)
Supplement: Supplementary file 1 — Supplementary Information [file 41598_2018_37256_MOESM1_ESM.pdf]

Ligand-induced conformational switch in an artificial bidomain protein scaffold

Corentin Léger, Thibault Di Meo, Magali Aumont-Nicaise, Christophe Velours, Dominique Durand, Ines Li de la Sierra-Gallay, Herman van Tilbeurgh, Niko Hildebrandt, Michel Desmadril, Agathe Urvoas, Marie Valerio-Lepiniec and Philippe Minard§

Supplementary information

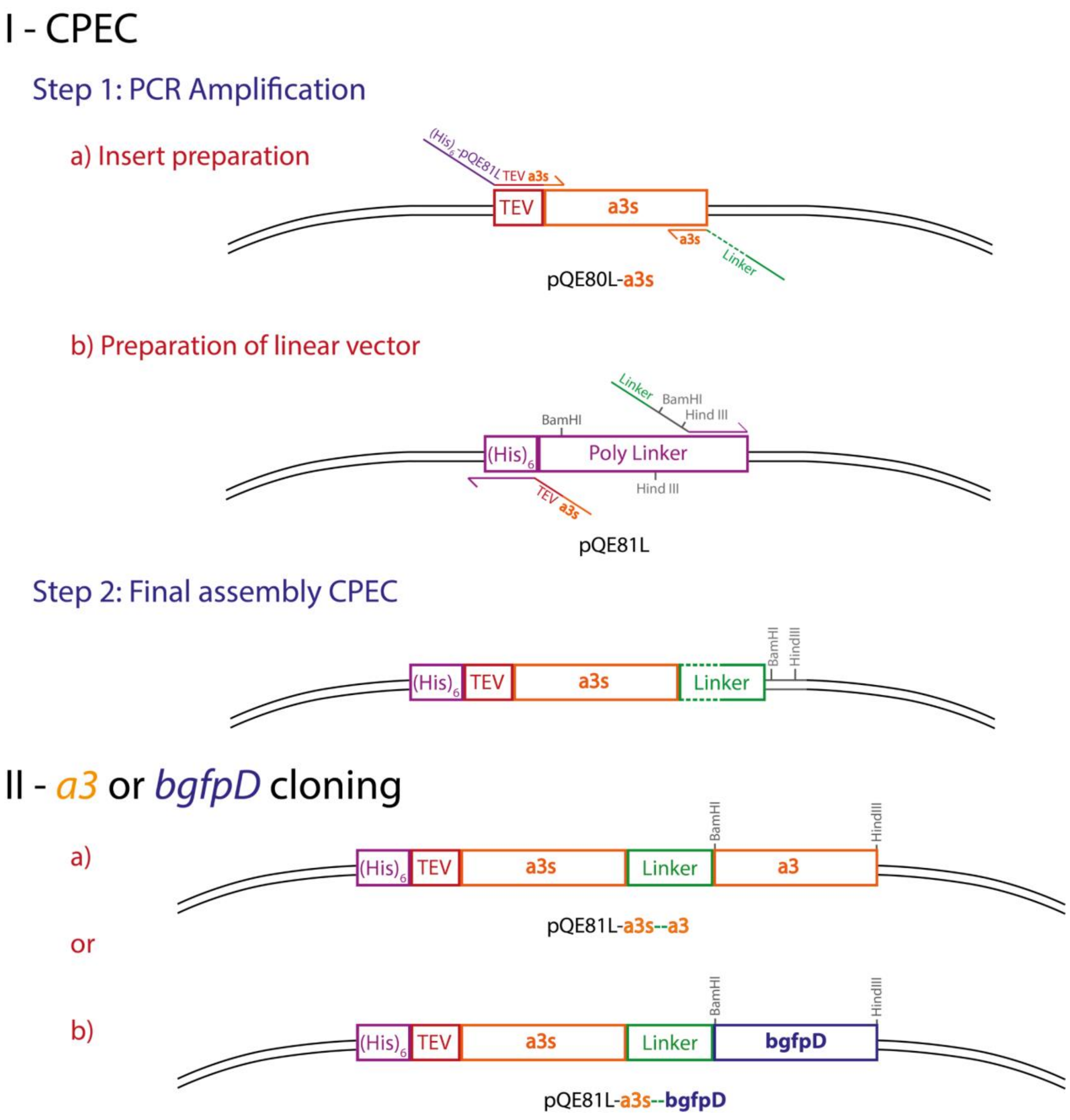

Figure S1: Genetic construction of bidomain A3_A3 and A3_bGFPD

The sequences coding for both bidomains, A3_A3 and A3_bGFPD, are cloned in the vector PQE81L. The construction coding for A3_A3 is composed of two distinct DNA sequences coding for the same αRep A3 protein. In the final bidomain both A3_A3 and A3_bGFPD proteins are separated by a linker $(SGGGG)_2$. The different steps of the construction are detailed below:

I- Construction of the gene "*a3s*_linker (SGGGG)$_2$ - new restriction sites BamHI/HindIII" in PQE81L using CPEC approach.

The primers designed to amplify the vector PQE81L and the gene insert "*a3s*" introduce overlapping regions as describe below.

Step 1a: Insert preparation:

The *a3s* insert was previously cloned in the plasmid PQE80L with a TEV protease sequence upstream the *a3s* gene (see material and methods). PCR1 is performed on the *a3s* sequence with the forward primer "His-PQE81L-*TEVa3s*" and the reverse primer "a3s----Linker".

The final product of this PCR is composed of the *TEVa3s* sequence flanked with a sequence that overlaps the PQE81L His-tag upstream, and a sequence that overlaps partially the linker sequence downstream the *TEVa3s* DNA sequence.

Step 1b: Preparation of linear vector:

The PCR on PQE81L vector with forward primer "Linker-BamH I/Hind III" and reverse primer "His-TEVa3s" corresponds to the following modifications:

The final product of this PCR corresponds to the linearized PQE81L vector where the restriction sites BamH I/Hind III from the multi-cloning site are removed. Moreover, a sequence overlapping a part of the linker followed by new sites BamH I/Hind III in 5', a sequence overlapping the TEV and a part of the *a3s* gene in 3' end are introduced.

Step2: Insertion of *a3s* in the adapted vector.

Final CPEC assembly and cloning reaction were prepared by mixing both PCR products (prepared linear vector and *a3s* inserts in molar ratio 1:1). This step consists in the hybridization of the overlapping regions between the insert and the adapted vector followed by PCR amplification with no primer added.

II Insertion of the sequence coding for A3 or bGFPD in the final CPEC vector.

The resultant product (PQE81L-His-TEVa3s-Linker-BamH I/Hind III) is double-digested with BamH I/Hind III; the *a3* gene, obtained by the digestion of the vector of the library with BamH I/Hind III, is then ligated into the gap (Figure S1-II -a). The gene coding for bGFPD was cloned using the same protocol (Figure S1-II -b).

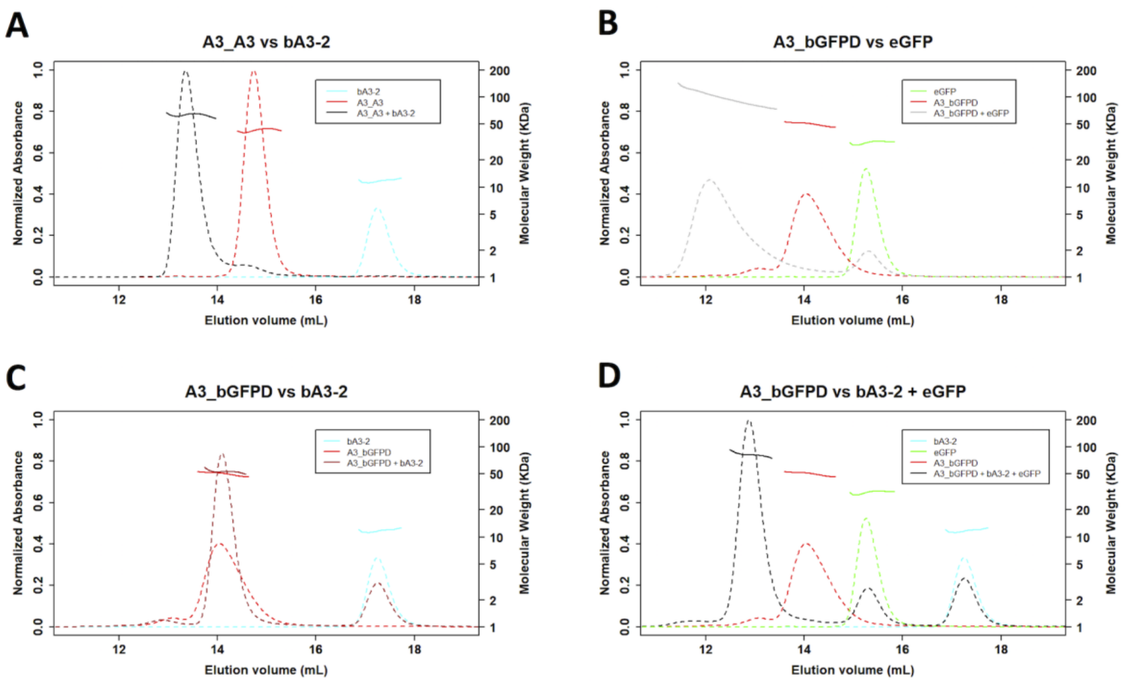

Figure S2. SEC-MALS elution profiles of bidomains A3_A3, A3_bGFPD and their complexes. Molecular Weight (hard lines) and Normalized absorbance (dash lines) are shown for eGFP, bA3-2, A3_A3 and A3_bGFPD. Purified proteins or mixtures (100 µL, 45 µM for all proteins except bA3-2 90 µM) were loaded on a Superdex 200 10/300GL column equilibrated in 50 mM NaP pH 7.5, 150 mM NaCl at a 0.5 mL/min flow rate and detected using a multi angle light scattering detector (Wyatts). For each eluted peak, the experimental molecular weight ($M.W_{exp}$), the molecular extinction coefficient (ε) and the hydrodynamic radius (Rh) were determined using the SEC-MALS Wyatt software. Profiles of the bidomain alone are represented in red, profiles of the binder bA3-2 in cyan, of the eGFP in green. The mix of the A3_A3 bidomain with the binder bA3-2 is represented in black, the mix of the A3_bGFPD bidomain with the eGFP in light grey, with the bA3-2 in brown and with both in black. For panels A, B, D, the shifts of the peaks for the bidomain to higher elution volumes in presence of their binders show that the bidomains are still able to interact with their ligand. For panel C, the isolated bidomain is in equilibrium between monomeric and dimeric forms (through interactions between A3 domains). bA3-2 binding induces both a shift of this monomer/dimer equilibrium and an increase of the A3_bGFPD apparent mass due to bA3-2 binding. No apparent shift of the main peak is therefore observed. The interactions of bA3-2 and A3_bGFPD can be quantified more precisely using the data obtained by refractometry allowing to calculate the number of moles of proteins in each peak. These data indicate that a fraction of bA3-2 (37 µg, 3.1 nmol) interacts with A3_bGFPD (137 µg, 3.1 nmol) to generate a new peak corresponding to the complexes (174.4 µg). For all proteins, the theoretical and experimental ε are comparable.

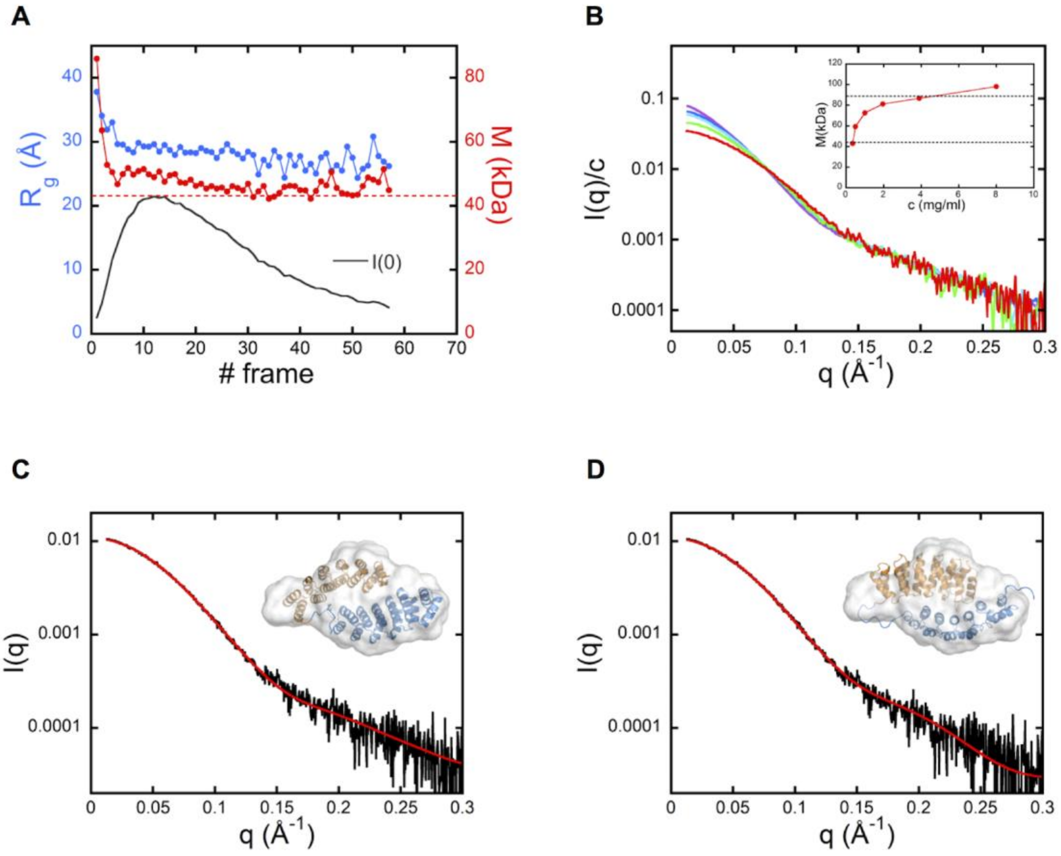

Figure S3: SAXS analysis of A3_bGFPD

S3-A: SEC-SAXS study of A3_bGFPD. Evolution with time of the radius of gyration Rg, of the forward scattering intensity I(0) and the molecular mass M. For each frame the acquisition time is 2s. The dashed horizontal line corresponds to the molecular mass of the monomer calculated from the sequence.

S3-B: Scattering intensities I(q)/c measured at different concentrations ranging from about 0.35 mg/ml to 8 mg/ml. The curve corresponding to the lowest concentration (red line) is the averaged scattering curve obtained on the SEC-SAXS beamline. The others curves were obtained by direct injection using the in-house SAXS instrument. The molecular masses shown in the insert were deduced from the SAXS curves using the program ScÅtter[1] available at the URL https://bl1231.als.lbl.gov/scatter/. The two horizontal lines correspond to the calculated molecular masses for the monomer and the dimer, respectively.

S3-C and S3-D: Analysis of the averaged SEC-SAXS curve measured at the end of the elution (black line). The red lines result of fitting the experimental curve using the program CRYSOL[2] from the atomic models shown in the inserts. In the model C the two domains A3 and b-GFPD are arranged as in the dimeric A3. The model D corresponds to the crystal structure of A3_bGFPD. Both fits are equally good (⍰^{2}=0.64 and 0.66, respectively). The light-grey colored envelope is deduced from the SEC-SAXS curve using the program *ab initio* GASBOR[3].

Note S1: SAXS methods and results

SAXS methods

SEC-SAXS data were collected at the BM29 line at the ESRF, Grenoble, with a size-exclusion HPLC column (Agilent Bio sec-3) online with a SAXS measuring cell (a 1.5 mm diameter quartz capillary in an evacuated sample chamber). This strategy is very useful to separate aggregates and/or oligomers from isolated proteins. The sample-to-detector distance was 2.867 m, covering the range of scattering vector (q) from 0.005 $Å^{-1}$ to 0.5 $Å^{-1}$. $q = 4\pi \sin\theta / \lambda$ where 2θ is the scattering angle and λ (0.992 Å) is the wavelength of the X-rays. The detector used was a Pilatus. The sample was kept at a constant temperature T = 20 °C. Scattered intensities were put on an absolute scale using water scattering. SAXS data were normalized to the intensity of the incident beam and background subtracted using the software IspyB[4] available on the beamline. The US-SOMO HPLC-SAXS module[5] was then used to determine the evolution of the radius of gyration Rg and of the molecular mass during the elution. Finally, an averaged SAXS curve was obtained by considering only the identical frames measured at the end of the elution.

Other SAXS experiments were performed on an in-house SAXS instrument (Brüker Nanostar; λ=1.54 Å). 30 µl of concentrated solutions (0.5 mg $mL^{-1} \leq c \leq$ 8.0 mg mL^{-1}) of A3_bGFPD were placed in a quartz capillary thermalized cell inserted into an evacuated sample chamber. SAXS data were analyzed using the program PRIMUS (https://www.embl-hamburg.de/biosaxs/primus.html).

SAXS results

In the first SEC-SAXS experiment, SAXS measurements were continuously performed while the proteins eluted from the size exclusion column. The sample concentration, monitored by a UV spectrophotometer installed before the SAXS cell, reached a value of up to about 0.5 mg mL^{-1} (12 µM). The radius of gyration R_g, the forward scattering intensity I(0) - which is proportional to the protein concentration - and the molecular mass M were extracted from each I(q) curve (Figure S3-A). M decreases strongly at the beginning of the elution, then slower, finally to reach the value of the molecular mass expected from the sequence, when the concentration is smaller than about 0.35 mg mL^{-1} (≈ 8µM). A simultaneous decrease of R_g is observed. A second SAXS experiment was performed by direct injection of concentrated solutions (0.5 mg mL^{-1} (12 µM) ≤ c ≤ 8.0 mg mL^{-1} (189 µM) into the SAXS cell. The scattering intensities I(q)/c normalized to the concentration change drastically when the concentration increases (Figure S3-B). A strong increase is observed in the small-q range indicating a monomer/dimer distribution shift in the 0,4-4 mg mL^{-1} (~10-100 µM) concentration range . The molecular masses extracted from each I(q) curve are shown in the insert. For the highest concentration (8 mg mL^{-1}, ≈ 200 µM) the value of M is even higher than the mass of the dimer.

SAXS references

1. Rambo, R. P. & Tainer, J. A. Accurate assessment of mass, models and resolution by small-angle scattering. *Nature* 496, 477–481 (2013).
2. Svergun, D. Determination of the Regularization Parameter in Indirect-Transform Methods Using Perceptual Criteria. *J. Appl. Crystallogr.* 25, 495–503 (1992).
3. Svergun, D. I., Petoukhov, M. V. & Koch, M. H. J. Determination of domain structure of proteins from X-ray solution scattering. *Biophys. J.* 80, 2946–2953 (2001).
4. Delagenière, S. *et al.* ISPyB: an information management system for synchrotron macromolecular crystallography. *Bioinforma. Oxf. Engl.* 27, 3186–3192 (2011).
5. Brookes, E., Vachette, P., Rocco, M. & Perez, J. US-SOMO HPLC-SAXS module: dealing with capillary fouling and extraction of pure component patterns from poorly resolved SEC-SAXS data. *J. Appl. Crystallogr.* 49, 1827–1841 (2016).

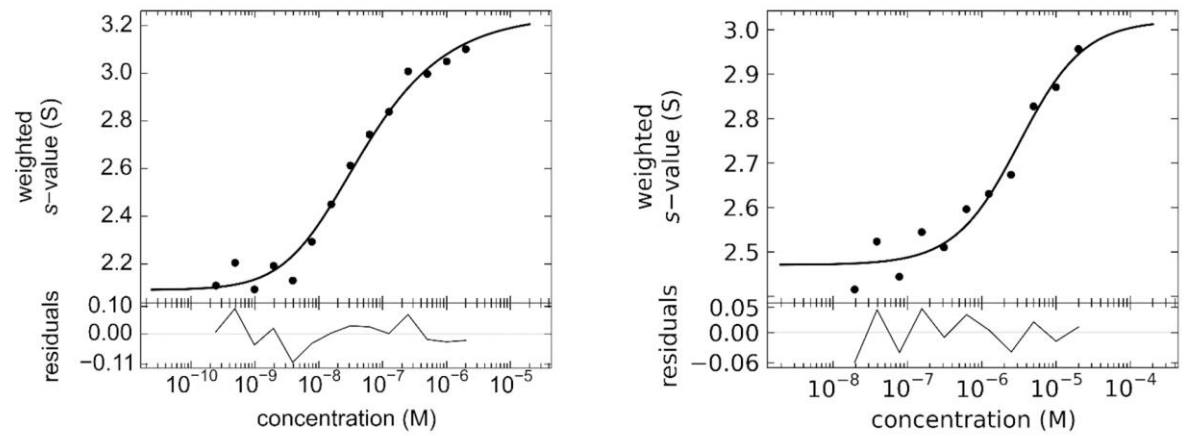

Figure S4. Analytical Ultracentrifugation analysis of the proteins dimerization.

(left) Determination of the dimerization constant of A3. Ultracentrifugation runs were performed using A3 concentrations ranging from 244 pM to 2 µM.

(right) A3 was incubated in the presence bGFPD: A3 concentration was fixed at 10 nM, bGFPD concentrations were from 19 nM to 20 µM.

In both cases, A3 was labeled with a fluorophore (Monolith NT-115 Nanotemper). Centrifugation was done at 128 297g at 20°C. Sedimentation velocity boundaries were analyzed using SEDFIT software (https://sedfitsedphat.nibib.nih.gov/software/default.aspx).

The resulting c(s) distributions were analyzed with GUSSI software (http://biophysics.swmed.edu/MBR/software.html) using sedimentation velocity isotherm mode. For each concentration, the signal weighted-average sedimentation coefficient (sw) was determined by integration and plotted (Analysis of high affinity self-association…). The sw isotherm was loaded into SEDPHAT (2) for fitting with the homo-dimerization model. The buffer density and viscosity at 20°C were determined by SEDNTERP software (T. Laue, Royal Society of Chemistry, Cambridge), partial specific volume for A3 was calculated based on the amino acid composition using this program.

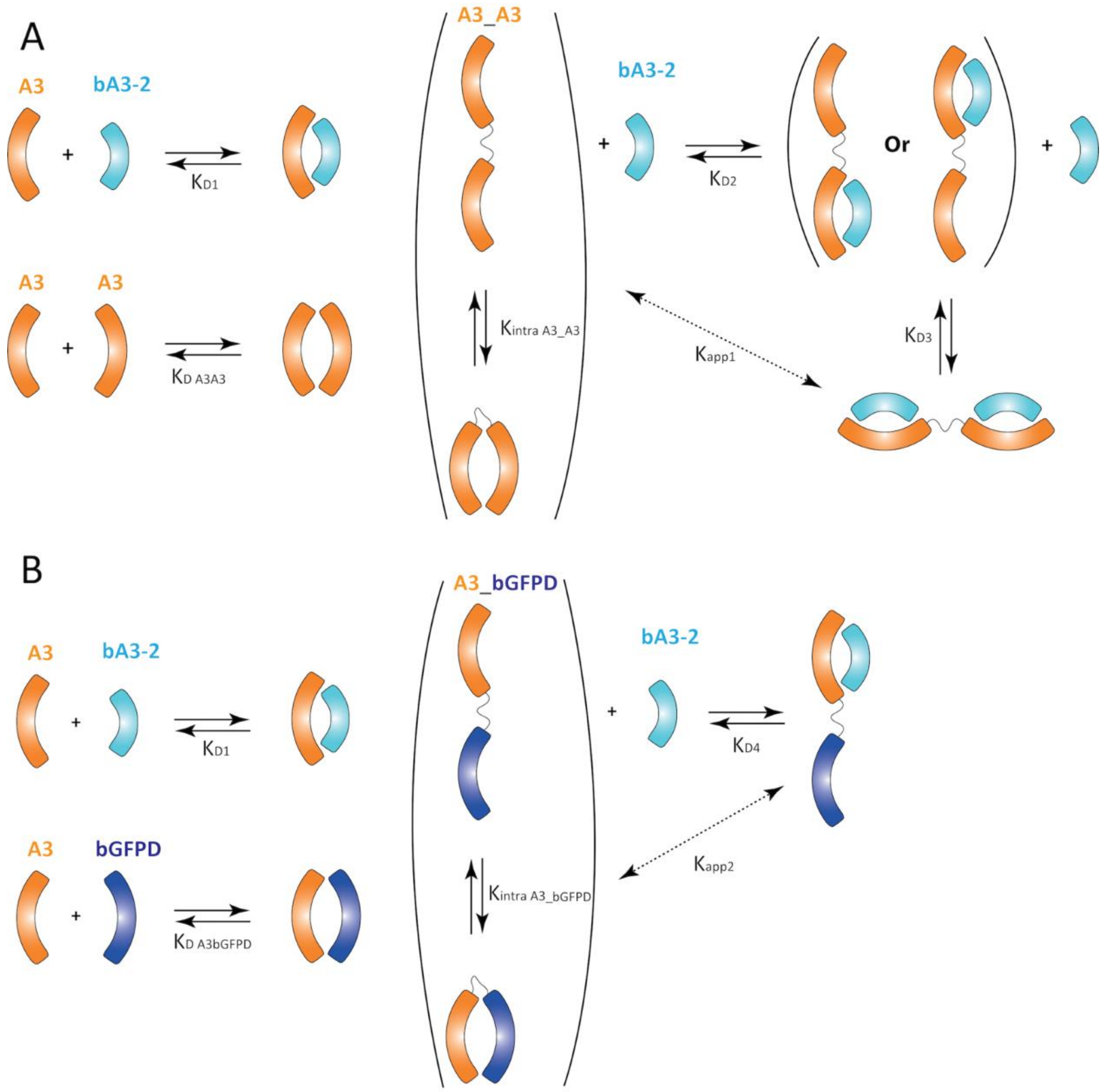

Figure S5. Models of the interactions in solution with the effects of the equilibrium between the open and closed conformations of the bidomains.

(A) Model of bA3-2 (in cyan) binding on A3 and on A3_A3 bidomain (in orange).

The dissociation constant of the A3A3 ($K_{D\ A3A3}$) homodimer was measured by UCA (Table 1). The dissociation constant K_{D1} for A3 in complex with bA3-2 was measured by SPR with a solution of momoneric A3 (below 2 nM). The apparent dissociation constant K_{app1} obtained by ITC, reflects the presence of the equilibrium between the open and the closed bidomain conformations driven by the constant $K_{intra\ A3\_A3}$. $K_{intra\ A3\_A3}$ is defined as the ratio of the A3_A3 concentration in the open form to the A3_A3 concentration in the closed form.

(B) Model of bA3-2 (in cyan) binding on A3 and on A3_bGFPD bidomain (in orange-blue).

The dissociation constant K_{D1} for A3 in complex with bA3-2 is the same as in (A). Although A3 and bGFPD are non-related αReps, they interact with low affinity and a micromolar

dissociation constant of the A3bGFPD heterodimer ($K_{D\ A3bGFPD}$) was measured by UCA and ITC (Table 1). The apparent dissociation constant K_{app2} for the binding of bA3-2 obtained by ITC, reflects the presence of the equilibrium between the open and the closed bidomain conformations driven by the constant $K_{intra\ A3\_bGFPD}$. $K_{intra\ A3\_bGFPD}$ is defined as the ratio of the A3_bGFPD concentration in the open form to the A3_bGFPD concentration in the closed form.

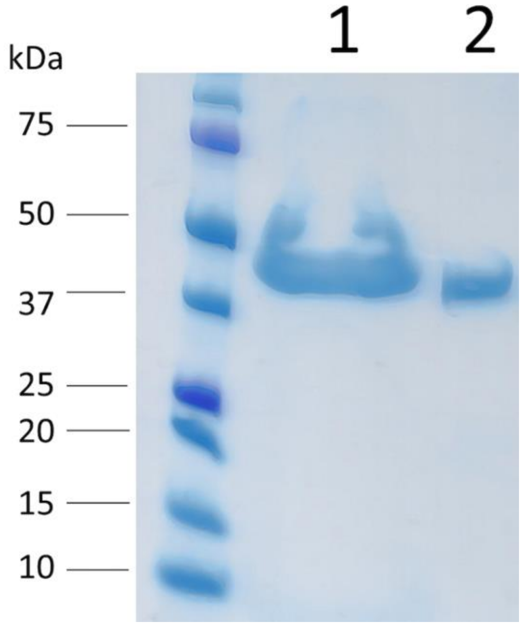

Figure S6: SDS-PAGE analysis of the crystallized A3_A3. Line 1: One crystal was washed three times in water and dissolved in the denaturation buffer. Line 2: Purified protein before crystallisation.

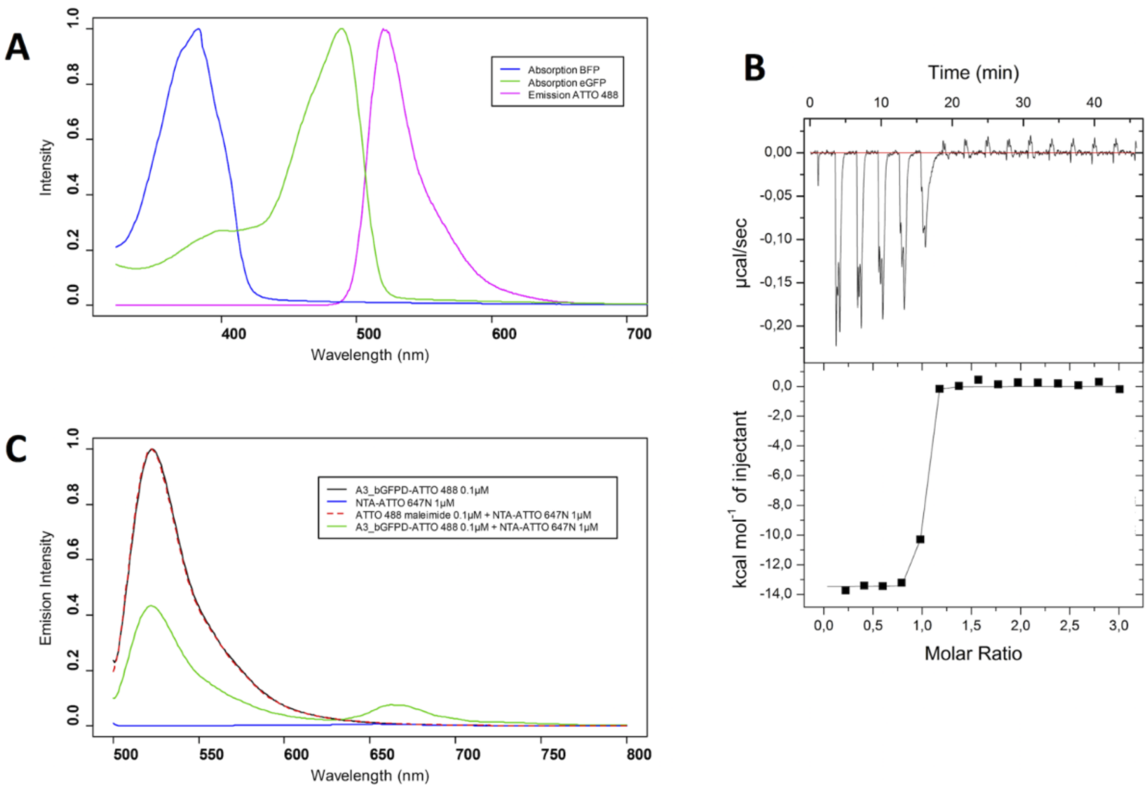

Figure S7: Control experiments for the FRET assays using A3_bGFPD

A) Normalized absorption spectrum of BFP (blue lines), eGFP (green line) and emission spectrum of ATTO 488 (pink line). Absorption spectra were obtained using a Perkin Elmer Lambda 35 spectrometer. Emission spectrum of the atto488 was obtained *via* the manufacturer website. There is and overlap around 500 nm between eGFP and ATTO 488. Therefore, the Blue fluorescent Protein (BFP), a variant of eGFP without spectral overlap was selected as a ligand in order to monitor FRET variations, without interferences due to the ligand.

B) ITC titration of a solution of BFP (15.7 µM) with bGFPD (300 µM). These data indicate that the BFP bind to bGFPd with the same affinity as eGFP. The stoichiometry n is equal to 0.94 ± 0.01, the K_D is 5 ± 2 nM.

C) Fluorescence emission spectra (excitation at 488nm) of A3_bGFPD-ATTO 488 (0.1 µM) (black line), NTA-ATTO 647N (1 µM) (blue line), ATTO 488 maleimide (0.1 µM) mixed with NTA-ATTO 647N (1µM) (red dashed line) and a mixture of A3_bGFPD-ATTO 488 (0.1 µM) and NTA-ATTO 647N (1 µM) (green line). The emission signal at 662 nm results from fluorescence transfer between the two dyes in the bidomain.

Table S1: Crystallographic data collection and refinement statistics.

Crystallographic data collection

	A3_A3	A3_bGFPD
X-ray source	ID29	ID29
Wavelength (Å)	0.97625	0.97
Temperature (K)	100	100
Unit-cell parameters (Å, °)	a = 89.65, b = 46.55, c = 63.21, α = 90.0, β = 128.93, γ = 90.0	a = 66.90, b = 88.90, c = 143.50, $\alpha = \beta = \gamma$ = 90.0
Space group	C2	$C222_1$
Resolution limits† (Å)	44.69-1.94 (2.06 -1.94)	47.87-2.55 (2.70-2.55)
Number of observations†	68413 (10144)	94784 (15058)
Number of unique reflections	15038 (2340)	14332 (2230)
R-meas† (%)	9.9 (79.6)	15.5 (173.9)
Completeness† (%)	97.6 (94.7)	99.7 (98.6)
I/σ† (I)	10.71 (1.94)	9.24 (1.09)
CC (1/2)	99.8 (87.5)	99.7 (82.2)

Refinement

	A3_A3	A3_bGFPD
Number of non-hydrogen atoms (protein/other)	1432/97	2829/8
R/R_{free} (%)	17.48/22.21	23.59/26.24
R.M.S.D. Bonds (Å)/angles (°)	0.018/1.855	0.008/0.947
Average temperature factors (protein/other)	44.47/61.80	68.71/65.91
Number of residues (asymmetric unit / chain)	183/409	368/409

† Values in parentheses refer to the highest resolution shell.

Accession number

Coordinates and structure factors have been deposited in the Protein Data Bank with accession number 6FT5 (for A3_A3) and 6HWP (for A3_bGFPD).
